# Rethinking the scale up of Integrated Management of Childhood Illness

**DOI:** 10.1136/bmj.k2993

**Published:** 2018-07-30

**Authors:** Smruti Patel, Jerome Pfaffmann Zambruni, Daniel Palazuelos, Hailemariam Legesse, Ndeye Fatou Ndiaye, Anne Detjen, Samira Aboubaker

**Affiliations:** 1 Washington, DC, USA; 2 Child Health Unit, United Nations Children’s Fund, New York, USA; 3 Partners in Health and Division of Global Health Equity, Brigham and Women’s Hospital, Harvard Medical School, Boston, USA; 4 Health Programs, United Nations Children’s Fund, Addis Ababa, Ethiopia; 5 United Nations Children’s Fund, Middle East and North Africa Regional office, Amman, Jordan; 6 Maternal, Newborn, Child Survival, and Adolescent Health and Development, WHO, Geneva, Switzerland

## Abstract

The global community must come together to rethink approaches and translate existing knowledge into action, say **Smruti Patel and colleagues**

**Key messages**

- Integrated Management of Childhood Illness (IMCI) has promoted as the service delivery model to reduce death, illness, and disability and to promote growth and development among children under 5 years of age
- To scale up effective coverage child health must become a matter of public concern, with commitment from all actors contributing to the health of the child
- Public health policy needs to shift from targeting the greatest number to targeting those most in need
- The pyramidal distribution of power within health system governance must change, putting citizens, communities, civil society, and frontline health workers at the top

Sick children have the right to quality medical care that restores them to greater health. Since the mid-1990s, Unicef and the World Health Organization have promoted Integrated Management of Childhood Illness (IMCI) as the comprehensive service delivery model at primary care level to achieve this goal. The full IMCI package involves three components: improving the case management skills of healthcare staff; improving overall health systems; and improving family and community health practices. After almost three decades of the implementation of these components across different contexts, the level of effective coverage varies between countries.

Based on the strategic review of IMCI undertaken by Unicef and WHO in 2016, we have analysed how the three components have been implemented. The experiences of different countries show the successes and constraints that influence IMCI implementation in different political, epidemiological, and social contexts. We identified three major determinants of effective coverage: political leadership to ensure an enabling environment; strengthened health systems based on empowered, recognised, motivated, supplied, and supervised frontline health workers; and empowered communities that can hold systems accountable and utilise IMCI services. A paradigm shift is needed to make sure that the key components of IMCI are implemented at scale.

## Background

IMCI has been associated with reductions in child mortality^1^ and has been introduced into more than 100 countries since the 1990s (box 1).^2^ Early adopters recognised the need to complement innovative integrated clinical management protocols with specific health systems strengthening efforts that are related to management and supplies,^3^ ^4^ as well as community based interventions to raise awareness around early care seeking and to improve family and community health behaviours.^5^ Subsequently, these three components were presented as a comprehensive package, accompanied by training tools and operational guidelines.

Box 1 **What is IMCI?**

In 1997, WHO and Unicef developed the Integrated Management of Childhood Illness (IMCI), a strategy that focuses on the right to heath and the wellbeing of the whole child.

IMCI aims to reduce death, illness, and disability, and to promote improved growth and development among children under 5 years of age.

IMCI includes both preventive and curative elements that are implemented by families and communities as well as by health facilities.

The strategy includes three main components: improving case management skills of healthcare staff; improving overall health systems; and improving family and community health practices.

World Health Organization www.who.int/maternal_child_adolescent/topics/child/imci/en.

Cohesive implementation of all three components has been identified as a critical factor in achieving high and sustained IMCI coverage. In Tanzania, IMCI training to improve the skills of health workers for better case management in health facilities—accompanied by health systems strengthening efforts such as improving supply of essential drugs—resulted in a 13% reduction in under 5 mortality over two years.^6^ In Bangladesh, a combination of community and health facility approaches resulted in substantial increases in service use.^7^

There has, however, been wide variability in achieving effective IMCI coverage. The 2016 IMCI implementation survey, conducted in 95 countries, showed that in 42% (31 of 74) of community level health facilities at least 60% of the health workers who cared for sick children were trained in IMCI.^8^

This paper focuses on the operationalisation of the three components of IMCI, and suggests ways to strengthen coordination between clinical care, service delivery, and community based activities to improve scale and effective coverage.

The findings presented in this article are based on three key sources: the 2016 Strategic IMCI Review, coordinated by Unicef and WHO, which describes lessons learnt from 20 years’ implementation of IMCI and recommends options for future child health strategies^2^ ^9^; a 2016 comprehensive review of the status of IMCI in the 21st century by Unicef that summarises scientific and programmatic evidence and IMCI health system aspects^3^ ^10^ ^11^; and the 2016 Global IMCI Implementation Survey, conducted among WHO member states and aimed at understanding the current implementation, challenges, strengths, and evolution of IMCI.^8^

## Critical factors for successful IMCI programming

**Fig 1:**
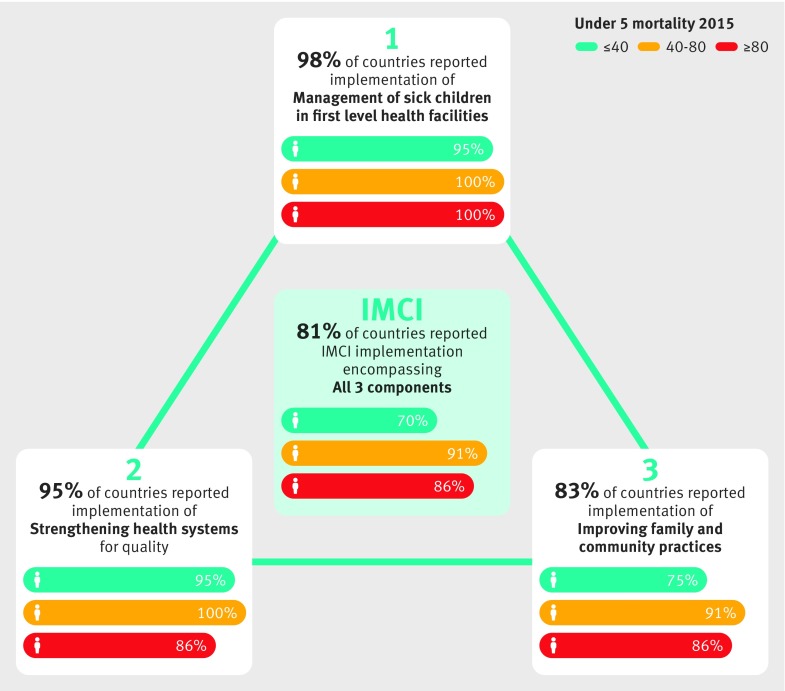
Proportion of countries reported to be implementing each IMCI component, disaggregated by level of under 5 mortality rate^8^

Fragmented IMCI leadership among global and country level partners, combined with uncoordinated and varying rates of development of materials and tools for each component, has limited the ability of countries to establish coherent, integrated IMCI programmes.^2^ Although most (81%) countries in the IMCI survey reported integration of all three components (fig 1), there is significantly lower implementation among countries with higher child mortality rates and high variations in the actual scale of implementation.^8^ A more thorough analysis shows significant challenges that need to be tackled to ensure effective implementation at scale.^11^

In some countries, the facility and community components have been introduced suboptimally and without coordination among implementing partners. In Peru, this resulted in the introduction of the facility and community components in separate districts with a loss of the expected synergy.^12^ Comparatively weak technical and financial support for the health systems strengthening component resulted in challenges for countries trying to align systems strengthening activities with the other components.

Four critical factors stand out for successful IMCI programming^2^: strong central leadership; commitment to strengthening health systems; clear vision and focus on integration between primary and community levels of care; and strong pre-existing community networks.

### Central leadership and commitment

Government led planning and ownership was identified as a primary factor for the success of integrated IMCI programmes at scale.^11^ In Egypt, initial and continued government commitment was evidenced by the establishment of a national, budgeted Ministry of Health IMCI programme. Advocacy efforts, including workshops for high level decision makers, resulted in programme institutionalisation. This engagement continued at district level with structured planning and orientation, as well as ensuring sufficient managerial capacity and health facility readiness to absorb new practices.^13^ IMCI implementation was associated with a doubling in the annual rate of reduction of under 5 mortality (3.3% *v* 6.3%) between 2000 and 2006.^13^

**Fig 2:**
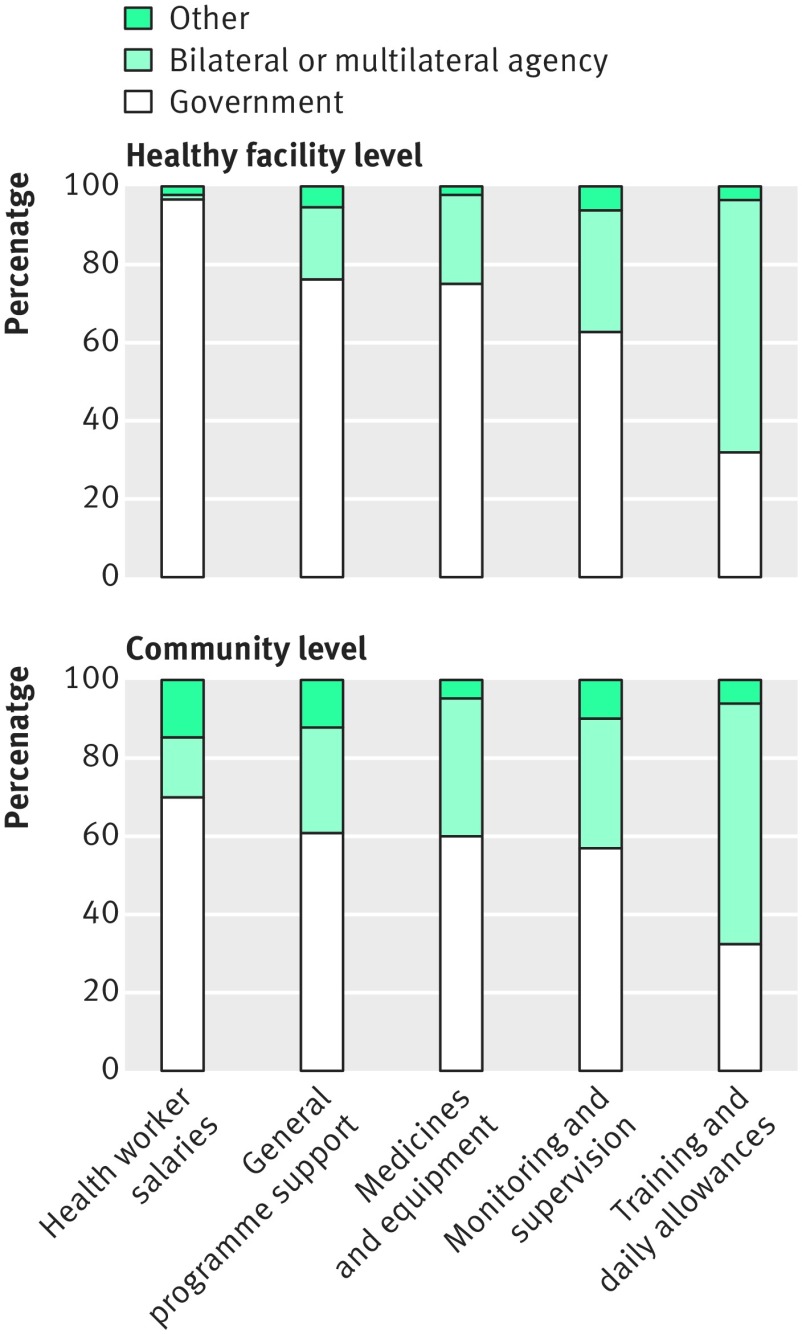
Main sources of funding for different IMCI implementation elements^8^

Assured domestic financing is equally important to show government commitment and leadership. Only 27.5% of countries reported that their government funds more than 75% of their IMCI programmes. Furthermore, 85% of countries with high levels of under 5 mortality (over 80/1000 live births) reported that their government funds less than 50% of their IMCI programme.^12^ Figure 2 presents the main sources of funding for IMCI implementation at health facility and community levels, showing that the community level especially relies heavily on donor support.

The investment needed for scaling up IMCI are often poorly defined and coordinated at global and national levels. IMCI generally starts as a donor driven initiative with a commitment to initial funding but not scale up. IMCI implementation survey respondents often reported insufficient budget for training (82.4%), cost of programme sustainability (59.8%), and lack of a dedicated budget line (63%) as barriers to implementation and scaling up.^8^ Interviews with global key informants suggests that IMCI’s lack of novelty and focus on short term goals negatively influenced long term funding.^2^ Egypt, however, shows how earmarked government funding can contribute to the success of long term IMCI at scale.^13^

### Strengthening health systems

Health systems affect IMCI implementation and scale up.^2^ ^10^ ^11^ The foremost problem is human resources, including insufficient quality and quantity of staff, turnover, and mobile workforce. At regional level, 84% of countries cited staff turnover, and at facility level 80% identified staff retention, as barriers to IMCI implementation.^8^ Pointing again towards a lack of coordination between components was the description of health facility based staff, not trained in IMCI, expected to supervise local volunteers performing community IMCI, and unpaid volunteers’ lack of motivation for community IMCI. Only 20.7% of low income countries provide a salary for their community health workforce.^8^ In one third of countries implementing IMCI, 75% of community level health facilities had at least 60% of health workers trained in IMCI. However, most countries with high under 5 mortality rates had less than 50% of facilities with health workers trained in IMCI, which impacts service delivery and outcomes for children.^8^

Lack of supportive supervision for health workers at central, regional, and facility level limit the effectiveness of training.^2^ For health workers in community level facilities, only 15.2% globally and 6.3% in countries with high under 5 mortality rate had had at least one supervisory visit in the past six months.^8^ Similarly highlighted were the lack of refresher training and continuous education. In order to reduce costs, 72% of countries incorporated IMCI into pre-service training of health professionals, 42% shortened the length of IMCI training, and 12% used the IMCI computerised adaptation and training tool in place of in-person training.^2^ Training problems are discussed elsewhere in this series.^14^

While essential equipment and consumables are mandatory for effective IMCI implementation, 48.9% of all countries, and 62.1% of low income countries, identified drug procurement and supply chain management as a barrier.^2^ This is a problem at all levels, but at the regional level, 60% of high mortality countries report it as a barrier, compared with only 29% of low mortality countries.^8^ Specific challenges include drug stockouts, poor quality drugs, lack of harmonisation in estimating needed inputs, and ageing facilities.

Lastly, robust monitoring systems are necessary to ensure that IMCI activities are implemented as planned and to assess whether the desired results are being achieved. Despite acknowledging the necessity of collecting IMCI related data, only a small proportion of countries have a comprehensive monitoring and evaluation plan for IMCI. Central databases are needed so that health facilities or district management teams with trained staff can provide regular reports, with built in feedback mechanisms from central to local levels.^2^

Niger exemplifies how many of the above mentioned health system problems can be effectively tackled.^15^ This high burden child mortality country introduced IMCI in 1996 and significantly reduced child mortality from 276 out of 1000 live births in 1996 to 96 of 1000 in 2015.^16^ Niger used the opportunity and resources brought by IMCI introduction to tackle long standing health systems weaknesses, such as human resources. A two tiered system of community health services, including formal salaried community health workers practising mainly at health post or village clinic level and a volunteer cadre operating at community or village and household level were introduced. These staff needed new training and enhanced supervision to ensure adequate skills for effective IMCI implementation. Changes in the policy environment provided an opportunity for community health workers to be formalised to promote access to basic healthcare services, and for the inclusion of a curative component to child health services. There has been a “task shifting” within primary healthcare, from clinic based clinicians to community health workers at the health posts. The volunteer cadres have a strong focus on health promotion and disease prevention. They work closely with both community health workers and clinicians in their catchment area, forming a crucial link between the community and health system. Volunteers receive training in essential family practices, stipends for training and campaigns, and equipment for their work.^15^

### Community empowerment and community-facility links

As well as providing an enabling environment with trained health workers and the necessary supplies, there needs to be a demand for IMCI. The IMCI implementation survey revealed that the community component of IMCI was initially neglected by many countries, though its importance was widely recognised.^8^ A 1999 progress report showed that “some countries are also implementing complementary IMCI activities to improve the health system and family and community practices.”^17^ In 2016, 83% of countries reported implementing the community component, but this proportion fell to 69.2% in countries where IMCI is not implemented at scale (less than 50% of districts).^8^ Global key informants thought that the design of IMCI did not include strong mechanisms to create demand for services at the community level.^2^ Successful mechanisms, in Niger for example, have included community dialogues and media messages (cinema, community radio, theatre) to disseminate information on promotion and prevention.^5^ Even where community IMCI did occur, it tended to be poorly defined with only patchy coverage of activities related to promoting key family practices. Community IMCI often appears separate from facility IMCI and supported by non-governmental organisations in isolation. In Ethiopia, the lack of formalisation of the volunteer cadre tasked to deliver community IMCI was identified as a factor contributing to its relatively poor implementation.^2^

Some have questioned whether community health workers s are able to deliver quality curative care, especially considering the potential consequences of inappropriate antibiotic use for antimicrobial resistance.^10^ However, ample studies have shown that community health workers are able to correctly assess and prescribe, if supervised and supported by the primary healthcare system.^18^ ^19^ Ethiopia has developed innovative mechanisms to support the community, whereby the Ministry of Health prioritised the implementation of community based preventive, promotive, and curative care through the health extension programme. As a result, the community component of IMCI received advanced support from both government and health development partners. Subsequently, the innovative Health Development Army (HDA) was introduced, training community level volunteers to focus on local behaviour change. The HDA is a network created between five households and one model family to influence each another in practising healthy lifestyles. To date, the government has been able to mobilise over three million women in the HDA.^20^ Since the 1997 adoption of IMCI, Ethiopia has reduced infant mortality from 162 in 1000 live births in 1997 to 59 in 1000 in 2015.

Countries that had already established a robust community health system were far more likely to take up and integrate the community IMCI component, as seen in Bangladesh where child mortality has declined from 98/1000 in 1997 to 38/1000 in 2015.^16^ Bangladesh has a longstanding history of providing community services. The international development organisation BRAC has demonstrated that community workers can promote and foster good family practices and respond to the needs of families and communities. A key factor is the daily presence of frontline health workers in villages. They have ongoing contact with mothers and families, allowing for access to the entire population, including those difficult to reach, while maintaining quality of care.^21^ Strong pre-existing links between community structures and the health system are a strong predictive factor of successful IMCI implementation. Possible future community engagement strategies are mentioned elsewhere in this collection.^22^

## Rethinking is necessary

Our analysis focused on key issues around governance, health systems, and community engagement that impact on successful IMCI scale up. We did not focus on technical challenges in clinical management protocols, gender inequalities, or humanitarian contexts. WHO is currently leading a revision of the technical protocols to tackle shifting epidemiology and lessons learnt.

Our findings suggest that, to improve child health, be sustainable, and scalable:

● IMCI needs to be implemented as part of a primary healthcare and community health system strengthening approach, guided by strong global and national political leadership
● Success relies on a supportive political and social enabling environment; a coordinated approach to planning and implementation led by national ministries of health (MOH) with support of global partners and donors; a sustainable and equitable service delivery system; and high utilisation of services based on policies that support demand and facilitate access to services
● IMCI service delivery across health programmes and partners needs to be harmonised under MOH leadership
● Integrated policies and support for frontline health workers need to ensure that the workforce is appropriately skilled, equipped, remunerated, and empowered to deliver high quality care for every child. This effort should be led by MOH, but include other sectors, such as finance and education
● Service delivery across the continuum of care and across the levels of the health system (from community to primary level to hospital care, inclusive of referral and counter-referral) needs to be institutionalised to ensure effective IMCI coverage
● Community empowerment and mechanisms supported by local and international partners as well as civil society—allowing caretakers to hold systems accountable for delivering quality IMCI—are key to ensuring demand, utilisation, and availability of services.

To secure the above points, the global community needs to come together to rethink approaches and translate existing knowledge into action.

Firstly, child health must become a matter of public concern, with commitment from all actors contributing to the health of the child.

Secondly, public health policy needs to shift from targeting the greatest number to targeting those most in need. Applying a systematic bias in favour of the poor will ensure that the three components of IMCI are made available for every child, everywhere. Another article in this collection discusses concrete ways for policy makers to focus on the poor.^23^

Thirdly, it is crucial to change the pyramidal distribution of power within health system governance.^24^ Currently, policy and implementation decisions are largely made by central government based on global policy and recommendations, with the expectation that these will trickle down to lower levels of the health system. Difficulties arise with this approach in the face of fragile political and administrative structures. It is time to change the perspective of the pyramid, and health policy making and implementation, by putting citizens, communities, civil society, and frontline health workers at the top—and then articulate the system and build policies to ensure that these health workers are empowered, within a decentralised health system, to deliver sustainable, affordable, acceptable, and quality services to every child, everywhere.

## Notes

See www.bmj.com/child-health for other articles in the series.

Contributors and sources: All authors contributed intellectual content, edited the manuscript, and approved the final version for submission. In addition, the authors would like to thank the many contributors to the research underpinning the 2016 Strategic Review of IMCI. The authors alone are responsible for the views expressed in this article, which does not necessarily represent the views, decisions, or policies of the institutions with which the authors are affiliated.

Provenance and peer review: Commissioned; externally peer reviewed.

This article is part of a series based on findings from a global review of Integrated Management of Childhood Illness (IMCI) funded by the Bill and Melinda Gates Foundation. Open access fees were funded by the Health Systems Research Unit, South African Medical Research Council. *The BMJ* peer reviewed, edited, and made the decision to publish the article with no involvement from the Medical Research Council.
